# Neurophysiological and Behavioural Effects of Combined Cannabis Concentrates and Alcohol Among People Who Regularly Use Alcohol and Cannabis

**DOI:** 10.1111/adb.70194

**Published:** 2026-10-27

**Authors:** Eleftherios M. Hetelekides, Patricia L. Davies, Mark A. Prince, William J. Gavin, Hollis C. Karoly

**Affiliations:** ^1^ Department of Psychology Colorado State University Fort Collins Colorado USA; ^2^ Department of Occupational Therapy Colorado State University Fort Collins Colorado USA; ^3^ Molecular, Cellular, and Integrative Neurosciences Program Colorado State University Fort Collins Colorado USA; ^4^ Department of Psychiatry and the Behavioral Sciences Keck School of Medicine Los Angeles California USA; ^5^ School of Music, Theatre, and Dance Colorado State University Fort Collins Colorado USA; ^6^ Department of Psychiatry, School of Medicine University of Colorado Anschutz Medical Campus Aurora Colorado USA

**Keywords:** cannabis, cognitive function, ecological study, EEG/ERP, performance monitoring, substance use

## Abstract

Increasingly potent concentrated cannabis products containing high levels of Δ^9^‐tetrahydrocannabinol (THC) are being consumed with alcohol. This combination is known to acutely impair cognitive and psychomotor abilities, although few studies have evaluated alcohol and cannabis co‐use using neural measures. This naturalistic feasibility study explored the acute effects of co‐intoxication on behavioural performance and brain activity, measured via electroencephalography (EEG), during a performance monitoring task administered three times over a 4.5‐h period. Participants (*N* = 17, 65% male) were heavy drinking community members (age M = 31.7 years; SD = 10.27) who regularly use cannabis. EEG was recorded while participants completed a speeded visual‐flanker task at baseline (sober) before consuming a standardized dose of alcohol and a cannabis concentrate product, followed by EEG recordings 1‐h (intoxicated) and 4‐h post co‐consumption (recovery). Recordings were processed to obtain six event‐related potential components: the stimulus‐locked N1, P2, N2 and P3 and response‐locked error‐related negativity (ERN) and error positivity (Pe). Repeated measures ANOVAs revealed that behavioural measures at the intoxicated timepoint showed an increase in errors (*p* < 0.005; *η* ^2^_*p*_ = 0.35) and significant decrease in response time (RT) (*p* < 0.001; *η* ^2^_*p*_ = 0.50) compared to sober and recovery periods. Measures of brain activity also demonstrated significant change across time with largest amplitudes when sober, smaller amplitudes when intoxicated, then an increase in amplitude to near sober levels at recovery for stimulus‐locked (P2, N2 and P3) and response‐locked components (ERN and Pe). Thus, compared to sober and recovery periods, the behaviour at the intoxicated timepoint revealed significantly greater impulsivity (RT) leading to significantly higher error rates, while measures of cognitive processing (i.e., decision‐making, error detection, performance monitoring/adaptation) showed significantly diminished levels of brain activity. Performance and neurophysiology measures remained diminished at recovery compared to baseline. Understanding neurocognitive functioning during co‐intoxication may provide insights into specific cognitive processes that may impact function in everyday activities such as driving.

**Trial Registration:** NCT06259916

## Graphical abstract

This naturalistic feasibility study explored the effects of alcohol and cannabis co‐intoxication on behavioural performance and brain activity using electroencephalography. A performance monitoring task was administered three times over 4.5‐h at sober, intoxication, and recovery timepoints. Compared to sober and recovery timepoints, behaviour during intoxication revealed greater impulsivity (faster responding) and increased errors, and cognitive processing showed significantly diminished brain activity. Understanding neurocognitive deficits during co‐intoxication may provide insights into specific cognitive processes that impact public safety‐related activities like driving.

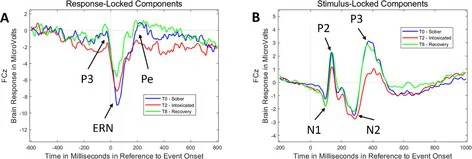

## 1 Introduction

Among populations reporting cannabis use, many also report using alcohol [1]. Research indicates that using alcohol and cannabis together, particularly simultaneously (i.e., such that intoxicating effects overlap), can lead to greater negative consequences compared to either substance used alone [2]. Co‐use of alcohol and cannabis can also increase subjective intoxication ratings and duration of intoxication [3], confer decrements in skills necessary for driving an automobile [4] and increase engagement in risky behaviours such as intoxicated driving [5]. Despite the risks associated with alcohol and cannabis co‐use, the existing laboratory research on this topic is limited, likely due to historical restrictions placed on cannabis research which have made it difficult for researchers to study the cannabis products that are proliferating on the legal market [6].

One important but understudied variable that likely impacts co‐use outcomes is the increasing Δ^9^‐tetrahydrocannabinol (THC, the primary psychoactive constituent of the cannabis plant) content available in the US cannabis supply. The few published studies that do provide laboratory‐based evidence on alcohol and cannabis co‐intoxication are limited by their use of low potency (i.e., 1%–11% THC) cannabis [7, 8], which is dissimilar from the legal‐market cannabis used by the US population today. The average THC content of contemporary cannabis flower available on the recreational market in Colorado is approximately 20%–25% [9]. Further, potent cannabis concentrate products (i.e., cannabis concentrates, dabs, wax, resins, etc.) contain upwards of 90% THC [10] and are increasingly available, especially in states with legal cannabis markets [11]. Cannabis products with greater levels of THC have been linked to a variety of negative consequences, including impaired cognitive functioning [12]. The growing availability and use of high‐THC cannabis products, along with the increasing prevalence of alcohol and cannabis co‐use, underscore the importance of understanding how these substances interactively affect brain activity and cognitive functioning. These outcomes can be assessed using neurophysiological tools like electroencephalography (EEG).

Growing interest in electrophysiological phenotypes of substance use disorders (SUDs), along with heterogeneity observed in event‐related potential (ERP) findings across substances, has led researchers to examine multiple ERP components (e.g., the P50, mismatch negativity, P300 and N400) in combination rather than in isolation. This approach posits that weighted combinations of electrophysiological features may better characterize substance‐specific neurocognitive profiles and differentiate both SUD‐related phenotypes and acute intoxication effects compared to a single ERP component approach (for a narrative review, see [13]).

Current research indicates that alcohol and cannabis may exert distinguishable effects on brain activity representing cognitive functions. Acute alcohol intoxication primarily depresses central nervous system activity, results in impairments in cognitive and motor performance [14], influences behaviour via actions across a variety of neurotransmitter systems [15] and has been found to regulate temporal patterns of resting‐state EEG networks [16]. Changes in specific ERP components induced by acute alcohol intoxication, such as reductions in the P3 [13] and error‐related negativity (ERN) components [17], have been reported. Further, alcohol intoxication has been shown to impact response conflict processing as well as impair cognitive and inhibitory control, evidenced by reduced P3 amplitudes, delayed P3 latency, diminished theta‐band activity and reduced ability to recover from, and neural responses during, error trials during a Flanker task [18, 19, 20, 21].

Cannabis is thought to confer a somewhat different pattern of effects on brain activity compared to alcohol. Driven primarily by actions of THC on the endogenous cannabinoid system and other diverse neurotransmitter systems [22], acute cannabis intoxication has been found to impair neurocognition, though these effects are likely dependent upon a variety of individual factors including biological, pharmacological, product formulation, frequency of use and recreational vs. medical use [23]. Research has observed decreased theta power [24], as well as diminished ERN, N4, late positive potential (LPP) and P3 ERP component amplitudes [25, 26] during acute cannabis intoxication. This may reflect cognitive disruptions relevant to performance impairments that were observed during a driving task in another study [27]. EEG measured changes in brain oscillations during intoxication have also been proposed as potential biomarkers of acute cannabis intoxication [24]. During the Flanker task specifically, previous research has observed dose‐dependent impairments in neural measures of performance monitoring, like the ERN and Pe [28, 29]. However, challenges related to interstudy variability, lack of effect size reporting, as well as heterogeneity of participant characteristics in the current body of literature preclude firm conclusions [26].

Altogether, current literature indicates that alcohol‐ and cannabis‐only intoxication diminish electrophysiological markers linked to inhibitory control and performance monitoring processes. These findings suggest that both substances may compromise neural systems underlying cognitive and inhibitory control, although potentially via distinct neuropharmacological mechanisms. Although substance‐specific effects on inhibitory control and performance monitoring have been established, how alcohol and cannabis interact to influence these processes during simultaneous co‐intoxication remains unclear.

To our knowledge, no studies have investigated the acute effects of simultaneous alcohol and cannabis co‐intoxication on brain activity using EEG/ERP measures. Additionally, although several EEG/ERP studies have assessed neural changes at multiple timepoints during acute intoxication, these measurements have largely been confined to the ascending or peak phases of intoxication (i.e., [30]). Comparatively few studies have systematically characterized behavioural and electrophysiological recovery across the descending limb of intoxication and into later post‐intoxication periods. Thus, despite compelling evidence suggesting potential additive or synergistic effects of co‐use on cognition, impairment and subjective outcomes [7, 8, 31] critical gaps in the literature remain.

The present feasibility study aimed to begin addressing these gaps by utilizing an ecologically valid, federally compliant, mobile laboratory approach. We observed ad libitum use of high‐THC cannabis concentrates from Colorado's legal market in combination with a standardized dose of alcohol. Using a within‐subject, repeated measures ABA design, we explored the acute effects of simultaneous intoxication by comparing participants at three timepoints over a 4‐h period (see Figure 1). This design allowed us to control interindividual variability as each participant was assessed first in a sober baseline condition (i.e., the control condition) then in an intoxicated condition and finally in a recovery condition after an extended time period post‐intoxication. We employed a performance monitoring paradigm (a speeded Flanker task) to assess performance‐related behaviours and associated ERP components. Performance monitoring is a critical cognitive function that underlies safe, goal‐directed behaviours, like driving and operating machinery. By assessing behavioural and neural indices of cognition and performance monitoring in a broad, exploratory fashion, we sought to capture global patterns of changes in brain activity related to safety and cognitive control across degrees of intoxication states.

**FIGURE 1:**
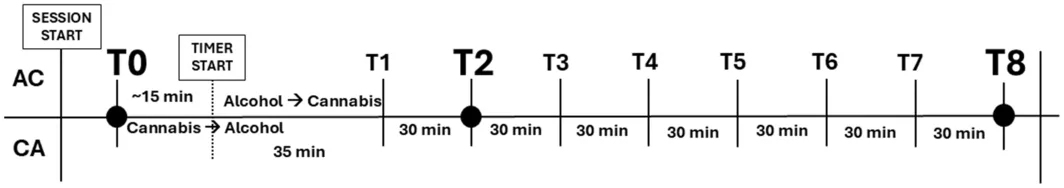
Timeline of data collection procedures within the study. *Note:* The timer began when participants started drinking their alcoholic beverage, regardless of whether they used cannabis before or after. Electrophysiological data and blood samples were collected only at Time 0 (T0‐ sober), T2 (intoxicated) and T8 (recovery, 4 h post‐alcohol onset).

Although ERP responses can vary across substances, tasks and experimental contexts, evidence from acute alcohol *or* cannabis intoxication studies has more consistently demonstrated attenuated neural responses during cognitive control tasks. Given the absence of prior studies examining ERP correlates of acute simultaneous alcohol *and* cannabis intoxication, we intentionally adopted a relatively broad and exploratory framework aimed at providing an initial characterization of intoxication‐related changes in stimulus‐ and response‐related neural activity, which will be useful for informing the design of future, large‐scale studies on this topic. Accordingly, we hypothesized that objective and subjective measures of intoxication, as well as performance‐related behaviours and ERP components, would display a systematic change such that, relative to the baseline and recovery timepoints, at the intoxication timepoint, participants would show the following:

1) greater levels of objective (breath alcohol content, blood plasma cannabinoid content) and subjective intoxication (the subjective high assessment scale),

2) worse behavioural performance on the task (assessed via number of errors committed, response times, post‐error slowing and variability of post‐error slowing behaviours), and

3) attenuated ERP component amplitudes (N1, P2, N2, P3, ERN and Pe).

While we expect these measures to show significant differences between their respective baseline and intoxication timepoints, the degree of recovery (i.e., comparisons of the intoxication timepoint to the recovery timepoint) may not be revealed as statistically significant if fatigue effects contribute to the measures given the length and intensity of the study's data collection procedures. However, if sufficient change back towards baseline values occurs, it should appear as a nonlinear (quadratic) relationship between the three timepoints. The degree of change, shown by the detection of a nonlinear effect, can be revealed in a test for a significant quadratic trend. Thus, a significant quadratic trend would provide evidence for changes in behavioural and brain activity measures following the timeline of the dissipation of the intoxicants in the participant's body and would indicate recovery of some function.

## 2 Methods and Measures

### 2.1 Participants

The present sample includes data from *N* = 17 individuals who reported heavy drinking and regular cannabis use, with at least one instance of cannabis concentrate use in the past year. Participants were between the ages of 21 and 56 (mean = 31.71, SD = 10.27) and primarily male (*n* = 11, 64.7%), White (*n* = 16, 94.10%) and non‐Hispanic (*n* = 13, 76.5%). Additional information about the sample can be found in Table 1.

**TABLE 1:** Descriptive statistics for the analytic sample (*N* = 17).

Categorical variable	Count	%
Sex		
Male	11	64.7
Female	6	35.3
Race		
White	16	94.1
Do not wish to respond	1	5.9
Ethnicity		
Hispanic	3	17.6
Not Hispanic	13	76.5
Do not wish to respond	1	5.9
Education		
Associates degree or technical certification	1	5.9
High school diploma or GED	3	17.6
Some college	8	47.1
Bachelor's degree	2	11.8
Master's degree	3	17.6
Order of drug consumption		
Alcohol first, then cannabis (AC)	8	47.1
Cannabis first, then alcohol (CA)	9	52.9

Continuous variable	M	SD
Age	31.71	10.27
Beck anxiety inventory (BAI)^2^	8.20	9.31
Beck depression inventory (BDI)	10.29	9.46
Marijuana dependence scale (MDS)	2.94	3.15
Alcohol use disorder identification test (AUDIT)^1^	13.50	6.01
Concentrates frequency (days per typical week)^1^	3.70	2.87
Flower frequency (days per typical week)^1^	5.51	2.42
Flower quantity (grams per typical session)^2^	0.27	0.38
Percent THC of product used^1^	76.11	7.38
Typical drinks per drinking day^1^	4.19	1.73

*Note:* Superscripts indicate differing sample size due to loss of data or participant refusal: ^1^ *n* = 16, ^2^ *n* = 15.

Participants were recruited from the community using flyers posted in public areas and online advertisements. Interested individuals were screened by lab personnel to determine their eligibility. The individuals whose data are presented in this report were recruited to participate in a smaller EEG feasibility substudy within a larger study and only details necessary to understand the EEG feasibility study are presented in this manuscript. This research study was approved by the University Institutional Review Board.

To be included in the study, individuals were required to report meeting the following criteria: (1) age 21–65; (2) meet heavy drinking criteria for at least the past 3 months, defined by the National Institute on Alcohol Abuse and Alcoholism (NIAAA) criteria (for males, consuming more than 4 drinks on any day or more than 14 per week and for females, consuming more than 3 drinks on any day or more than 7 per week); (3) regularly using legal‐market cannabis (at least 3×/week for at least the past 3 months); (4) report using a cannabis concentrate product at least once in the past year; and (5) willing to travel to a cannabis dispensary and purchase any cannabis concentrate product of their choice for use during the study.

Potential participants were excluded from the study if they reported the following: (1) being diagnosed with or seeking treatment for any SUD, (2) illicit recreational drug use in the past 60 days or failing the initial breathalyser or drug urinalysis (drug screen during the mobile lab session tests for the presence of amphetamines, benzodiazepines, cocaine, methamphetamine, opioids and THC, though THC was allowed), (3) having a major medical condition contraindicating alcohol and/or cannabis consumption (e.g., liver disease, heart disease, or being told by a doctor that it is unsafe to consume alcohol or cannabis due to a medical condition), (4) meeting criteria for, or having a history of bipolar disorder, psychotic disorder or major depressive disorder paired with suicidal ideation, (5) taking any psychotropic (besides anti‐depressants) or steroid‐based medications and (6) being pregnant, breastfeeding or trying to become pregnant.

### 2.2 Procedures

Prior to the mobile lab session, eligible participants were asked to complete a baseline survey hosted by Qualtrics (Copyright 2024 Qualtrics, Provo, UT, USA). This survey contained measures of demographics, substance use and psychological functioning necessary for characterizing the sample (see Table 1). Once participants completed the baseline survey, they were scheduled for a mobile laboratory visit at their residence.

The majority of the study took place inside the mobile laboratory parked outside the participant's residence with the exception of cannabis use, which took place inside the participant's home while connected with researchers via video call. As depicted in Figure 1, EEG data were collected at three timepoints: while participants were sober (T0), intoxicated (T2) and recovering from intoxication (T8). Participants were randomly assigned to consume either alcohol or cannabis first, such that half of participants consumed alcohol first and then cannabis (AC) and half consumed cannabis first and then alcohol (CA).

#### 2.2.1 Activities at Each Timepoint

At all timepoints, data were collected on a variety of survey questions via Qualtrics (see Section 2.3 below) and breathalyser assessments of Breath Alcohol Content (BrAC). At T0, T2 and T8 only, participants completed a series of tasks while brain activity was recorded via EEG, and blood samples were drawn by a research assistant trained in phlebotomy. The three timepoints for collecting EEG were chosen to reflect when we expected participants to reach peak BrAC (~1‐h post use; [32]) and recover from use (~4‐h post use; [33]).

#### 2.2.2 Substance Use During the Mobile Lab Session

Participants were administered a standardized amount of vodka based on the Widmark formula [34] to target a blood alcohol concentration of approximately 0.08 g/dL. They were asked to consume the drink within 20 min but not before 10 min had elapsed after taking their first sip. Participants were offered orange juice as a mixer and could choose the amount of orange juice they wanted to use. Alcohol administration procedures occurred inside the mobile laboratory.

For all participants, cannabis self‐administration occurred inside their residence, while researchers remained in the mobile lab. Participants were instructed to use their cannabis product ad libitum in the way they normally use it or to use however much they felt comfortable using. Researchers established a video call with participants connected on their own device, and participants were then sent inside their residence without the researchers to prepare their cannabis concentrate product for use. Participants then used their cannabis product ad libitum, in the way they normally do (i.e., using a dab rig, cannabis concentrate vaporizer or other method of consumption, consuming as much or as little of the product that they preferred) while maintaining visibility on the video call. After they finished smoking, participants provided the research team with information from the original dispensary packaging of the cannabis product that they had just used (e.g., product name, THC%) and returned to the mobile lab.

Participants were instructed not to consume alcohol, cannabis or any other psychoactive drug like caffeine or nicotine, for the remainder of the session. They were allowed to leave the mobile lab to attend to any urgent needs (e.g., to use the restroom) but were asked to remain in the mobile lab with research personnel for as much of the session as possible.

### 2.3 Survey Measures

Participants completed survey measures assessing demographics, anxiety (Beck Anxiety Inventory; [35]), depression (Beck Depression Inventory II; [36]), cannabis dependence (Marijuana Dependence Scale; [37]), alcohol dependence (Alcohol Use Disorder Identification Test; [38]) and frequency and quantity of cannabis and alcohol use (Table 1). At all timepoints, participants also completed the Subjective High Assessment Scale (SHAS; [39]). The SHAS includes items such as “High” and “Drunk or intoxicated” and asks participants how much these items describe their current feelings on a scale from 1 = *no alcohol effect* to 10 = *maximum alcohol effect*. See Supporting Information for more information on survey measures.

### 2.4 Electrophysiological paradigm

At each of the three EEG timepoints (T0, T2 and T8), participants completed the speeded visual flanker task [40]. The flanker task was not completed at any other study timepoints besides T0, T2 and T8. The task contained 480 total trials, presented in two blocks of 240. Participants were presented in rapid succession in one of four different stimulus types which were character arrays consisting of five letters. Stimuli included congruent stimuli (“HHHHH” and “SSSSS”, 80 trials each for 160 total trials) and incongruent stimuli (“HHSHH” and “SSHSS”, 160 trials each for 320 total trials). The stimulus duration was 250 ms. As the participant engaged with the task, the program evaluated the overall error rate every 30 trials, and if the error rate was greater than 25% or less than 10%, the interstimulus interval (ITI; initially set to 1400 ms) was increased or reduced by 100 ms, respectively. A minimal ITI of 800 ms was set to allow adequate time for processing the stimulus and behaviour prior to the onset of the following trial. Thus, task speed was customized to individual performance while ensuring an adequate error response rate for ERP analysis.

Participants were seated with their index fingers lightly touching the outermost response buttons on a five‐button keypad while the task was presented by E‐prime 3.0 [41]. The instructions were to press the left button using their left index finger if the middle letter of the string was H and to press the right button using their right index finger if the middle letter was S. Participants were informed that letters would be presented quickly. They were asked to perform as accurately as possible.

### 2.5 Blood Cannabinoids

After the mobile lab session, plasma was extracted from the three blood samples. Plasma was stored at −80°C until the completion of the study, when all plasma was analysed for cannabinoid content in a single batch. High‐performance liquid chromatography–tandem mass spectrometry was used to assay plasma concentrations of THC, 11‐Hydroxy‐Δ^9^‐tetrahydrocannabinol [11‐OH‐THC] and 11‐Nor‐9‐carboxy‐Δ^9^‐tetrahydrocannabinol [THC‐COOH] levels [42, 43, 44].

### 2.6 Electrophysiological Recordings and Data Reduction

EEG scalp recordings were collected for each administration of the speeded visual flanker task using a 64‐channel BioSemi ActiveTwo system (BioSemi Inc., Amsterdam, the Netherlands) with electrode placement based on the 10–20 system (American Electroencephalographic Society). Additionally, two bipolar electrooculograms (EOGs) were used to measure vertical and horizontal eye movements. Specifically, the vertical EOG (VEOG) was derived from two electrodes placed on the supra‐ and infraorbital regions of the left eye, while the horizontal EOG (HEOG) was derived from two electrodes placed on the left and right outer canthi. Electrodes were placed on each ear lobe to serve as the offline reference. The EEG recordings were collected at a sampling rate of 2048 Hz and downsampled to 1024 Hz for processing in Brain Vision Analyser 2.2 software (www.brainproducts.com). All scalp channels were referenced to the averaged signals of the bilateral earlobe channels then filtered with a bandpass filter of 0.1–30 Hz (24 dB/oct). Two collections of ERPs segments were generated for each participant: one time‐locked to the presentation of the stimulus for correct trials only and one time‐locked to the button press of incorrect trials only. Stimulus‐locked trials were segmented from −200 to 1000 ms followed by baseline correction using −200–0 ms. Response‐locked trials were segmented from −600 to 800 ms followed by baseline correction using −600 to −400 ms [45]. Eye movement artefacts were removed via a regression‐based approach using the VEOG channel [46] and baseline corrected. Segments with response times faster than 100 ms were excluded from analysis as were any segments with amplitudes greater than 100 μv or less than −100 μv. The average number of segments used in analyses for stimulus (correct trials) and response (incorrect trials) locked trials across the three timepoints are reported in Table 2.

**TABLE 2:** Descriptive statistics for outcome variable measurements (*N* = 17).

	T0—sober	T2—intoxicated	T8—recovery
Objective and subjective intoxication	M (SD)	M (SD)	M (SD)
Breath alcohol concentration (BrAC)	0 (0)	0.0684 (0.0122)	0.0176 (0.0122)
Subjective high assessment scale (SHAS)	15.00 (0)	54.36 (25.24)	25.19 (12.36)
Plasma levels of THC	11.04 (10.94)	42.84 (37.75)	12.33 (10.00)
Plasma levels of 11‐OH‐THC	5.36 (7.64)	12.23 (13.32)	5.77 (7.60)
Plasma levels of THC‐COOH	137.92 (208.85)	141.21 (181.90)	114.23 (174.94)
Behaviour data			
Number of correct trials	449.18 (19.22)	436.88 (30.81)	444.18 (25.67)
Number of errors (incorrect trials)	27.06 (17.62)	39.76 (28.44)	32.71 (23.78)
Response time (ms)			
Correct	440.33 (43.73)	407.49 (44.90)	415.85 (48.80)
Incorrect	379.87 (48.87)	353.88 (45.56)	370.35 (57.17)
Mean of post‐error slowing (ms)	96.38 (63.23)	52.65 (46.92)	36.22 (47.42)
Standard deviation of post‐error slowing (ms)	151.75 (90.48)	120.14 (53.28)	125.06 (66.98)
Frontal (FCz) ERP data (amplitude μV)			
ERN^a^	−12.08 (6.43)	−8.25 (3.88)	−9.04 (4.19)
Pe^a^	12.78 (5.60)	9.30 (3.24)	10.47 (3.20)
N1	−1.88 (1.66)	−1.94 (1.68)	−1.96 (1.30)
P2	4.95 (1.92)	4.49 (2.02)	5.50 (2.13)
N2	−6.81 (2.66)	−6.16 (2.57)	−6.64 (2.70)
P3	7.61 (3.33)	6.05 (3.22)	7.07 (3.10)
Posterior (P7, O1) ERP data (amplitude μV)			
N1 (P7)	−7.92 (4.73)	−7.33 (4.39)	−8.51 (4.59)
N1 (O1)	−9.38 (3.41)	−8.19 (3.61)	−9.49 (3.84)

^a^ Indicates ERPs where one individual did not commit a sufficient number of errors to be included in response‐locked analyses, and therefore, *n* = 16.

The averaged ERPs for each participant were scored via a custom peak picking software [47] that performs automatic scoring and provides visual inspection and manual correction of any values initially chosen on a slope instead of a peak. All components were scored at FCz and reported as peak‐to‐peak measures consistent with Gavin and colleagues (2019) recommendations. The windows used for scoring the frontal electrode ERP peaks were as follows P1 = 0–100 ms, N1 = 70–150 ms, P2 = 110–240 ms, N2 = 170–350 ms, P3 = 320–575 ms, ERN = −10–180 ms and Pe = 100–300 ms. In addition, the N1 was scored and analysed at posterior electrodes (P7, P8, O1 and O2) consistent with traditional methods of analysing N1 for visual paradigms [48]. The maximal negativity at posterior electrode sites was at P7 and O1; thus, data for P7 and O1 will be reported. The windows used for scoring the posterior electrodes ERP peaks were as follows: P1 = 80–120 ms and N1 = 130–185 ms. Previous literature has suggested strong test–retest reliability for stimulus‐locked and moderate‐strong reliability for response‐locked ERP components measured in neurotypical adults [49].

### 2.7 Statistical Analysis

Analyses were conducted using SPSS (v28) and R statistical software (v2023.03.0 + 386). The following packages were utilized in R: tidyverse [50], psych [51] and pwr [52]. Because individuals were prohibited from participating if they arrived with a BrAC above zero, BrAC exhibited zero variance at T0. This was also true for the SHAS, where participants were instructed to indicate a ‘1’ for each item at T0 (prior to alcohol consumption). Changes from T0 to T2 and T0 to T8 were evaluated using a one‐sample *t*‐test where values for BrAC and SHAS at T0 were set at an obtained mean of 0 for BrAC and a hypothesized mean of 15 for SHAS. A one‐sample *t*‐test was used because there was no variability at T0 for both BrAC and SHAS. Timepoints T2 and T8 were compared using a paired samples *t*‐test.

For blood cannabinoids (plasma THC, 11‐Hydroxy‐Δ^9^‐tetrahydrocannabinol [11‐OH‐THC], and 11‐Nor‐9‐carboxy‐Δ^9^‐tetrahydrocannabinol [THC‐COOH]) and behavioural data (number of errors, mean and standard deviation of post‐error slowing), a one‐way Repeated Measures Analysis of Variance (RMANOVA) with single factor timepoints (T0 vs. T2 vs. T8) was used to assess changes across time. A two‐factor ANOVA (timepoints: T0 vs. T2 vs. T8 and correctness of trial: correct vs. incorrect) was used to analyse differences in response times. Differences in peak‐to‐peak amplitudes across timepoints/intoxication states were tested on all ERP components separately using one‐factor RMANOVA (timepoints: T0 vs. T2 vs. T8). To control the familywise error rate, the Benjamini–Hochberg correction was used to adjust the critical *p*‐values for each test statistic [53]. On each of these repeated measures ANOVAs described above, rather than conduct pairwise post hoc tests to examine differences between timepoints, we examined quadratic trend effects to determine whether there is a departure of T2 from T0 and T8 on each dependent measure as hypothesized. Because blood THC data were available for only a subset of participants and data were systematically missing from earlier participants, statistical models tying substance levels to outcome variables were not feasible due to low power. Accordingly, BrAC and blood cannabinoid concentrations are presented descriptively, and analyses presume that the intoxication state is causally related to the observed changes in outcome measures.

## 3 Results

Descriptive statistics are reported in Table 1. Means and standard deviations on measures of objective and subjective intoxication, behaviour, and ERP components from the flanker task at each timepoint are reported in Table 2. Results of hypothesis testing on objective and subjective measures of intoxication, behavioural and ERP measures across the three timepoints of the study are reported in Table 3 and summarized in the following sections. Post hoc power analyses indicated that power for individual statistical tests ranged from 0.05 to 1, with the majority of tests powered at > 0.8. Results for the power of each test are shown in Table 3.

**TABLE 3:** Main effects of timepoint and quadratic trend effects on outcome variables.

Objective and subjective intoxication	*t/F* value	*df*	*p*	Effect size	Observed power
Breath alcohol concentration (BrAC)					
T0 vs. T2	22.94	16	< 0.001*	*d* = 5.56	1
T0 vs. T8	5.78	15	< 0.001*	*d* = 1.45	1
T2 vs. T8	19.50	15	< 0.001*	*d* = 4.88	1
Subjective high assessment scale (SHAS)					
T0 vs. T2	5.84	13	< 0.001*	*d* = 1.56	1
T0 vs. T8	3.298	15	0.005*	*d* = 0.82	0.87
T2 vs. T8	4.74	12	< 0.001*	*d* = 1.32	0.99
Plasma levels of THC	14.06	2,24	0.003*	*η* ^2^_*p*_ = 0.54	0.93
Quadratic trend effect	14.15	1,12	0.003*	*η* ^2^_*p*_ = 0.54	0.93
Plasma levels of 11‐OH‐THC	14.15	2,24	0.003*	*η* ^2^_*p*_ = 0.54	0.94
Quadratic trend effect	14.27	1,12	0.003*	*η* ^2^_*p*_ = 0.54	0.93
Plasma levels of THC‐COOH	3.81	2,24	0.037	*η* ^2^_*p*_ = 0.24	0.64
Quadratic trend effect	2.73	1,12	0.125	*η* ^2^_*p*_ = 0.19	0.33
Behaviour data					
Number of errors (incorrect trials)	6.79	2,32	0.003*	*η* ^2^_*p*_ = 0.30	0.89
Quadratic trend effect	8.65	1,16	0.01*	*η* ^2^_*p*_ = 0.35	0.79
Response time					
Correctness (correct vs. incorrect) main effect	93.70	1,16	< 0.001*	*η* ^2^_*p*_ = 0.85	1
Timepoint (T0 vs. T2 vs. T8) main effect	10.96	2,32	< 0.001*	*η* ^2^_*p*_ = 0.41	0.99
Quadratic trend effect	15.86	1,16	0.001*	*η* ^2^_*p*_ = 0.50	0.96
Mean of post‐error slowing (ms)	8.75	2,32	< 0.001*	*η* ^2^_*p*_ = 0.35	0.96
Quadratic trend effect	1.40	1,16	0.254	*η* ^2^_*p*_ = 0.08	0.20
Standard deviation of post‐error slowing (ms)	2.04	2,32	0.161	*η* ^2^_*p*_ = 0.11	0.33
Quadratic trend effect	1.42	1,16	0.250	*η* ^2^_*p*_ = 0.08	0.20
Frontal (FCz) ERP data (amplitude μV)					
ERN main effect	8.12	2,32	0.007*	*η* ^2^_*p*_ = 0.34	0.94
Quadratic trend effect	6.90	1,16	0.018*	*η* ^2^_*p*_ = 0.30	0.69
Pe main effect	4.76	2,32	0.028*	*η* ^2^_*p*_ = 0.23	0.75
Quadratic trend effect	4.69	1,16	0.046	*η* ^2^_*p*_ = 0.23	0.53
N1 main effect	0.15	2,32	0.859	*η* ^2^_*p*_ = 0.01	0.07
Quadratic trend effect	0.037	1,16	0.850	*η* ^2^_*p*_ = 0.002	0.05
P2 main effect	12.89	2,32	< 0.001*	*η* ^2^_*p*_ = 0.45	0.99
Quadratic trend effect	14.40	1,16	0.002*	*η* ^2^_*p*_ = 0.47	0.95
N2 main effect	3.67	2,32	0.037	*η* ^2^_*p*_ = 0.19	0.63
Quadratic trend effect	8.82	1,16	0.009*	*η* ^2^_*p*_ = 0.36	0.80
P3 main effect	10.63	2,32	< 0.001*	*η* ^2^_*p*_ = 0.40	0.98
Quadratic trend effect	16.09	1,16	0.001*	*η* ^2^_*p*_ = 0.50	0.96
Posterior (P7, O1) EPR data (amplitude μV)					
N1 main effect for P7	6.60	2,32	0.004*	*η* ^2^_*p*_ = 0.29	0.88
Quadratic trend effect	12.45	1,16	0.003*	*η* ^2^_*p*_ = 0.44	0.91
N1 main effect for O1	7.88	2,32	0.002*	*η* ^2^_*p*_ = 0.33	0.93
Quadratic trend effect	11.02	1,16	0.004*	*η* ^2^_*p*_ = 0.40	0.88

*Note:* Raw *p*‐values are reported numerically above, while the significance notation * indicates tests that met adjusted significance criteria (a false discovery rate of 0.05) following the Benjamini–Hochberg correction for multiple tests. The obtained critical value for the 37 tests conducted was equal to 0.037.

### 3.1 Intoxication and Behaviour Results

#### 3.1.1 Intoxication

Objective (BrAC and blood plasma levels of THC, 11‐OH‐THC and THC‐COOH) and subjective measures of intoxication (SHAS) were measured across time. Paired samples *t*‐tests were conducted for BrAC and the SHAS to examine differences in the sample across timepoints. Changes in BrAC over the course of the session are shown in Figure 2. *T*‐tests revealed that BrAC and SHAS measurements significantly differed across T0, T2 and T8 (Table 3 and Figures 2 and 3, respectively). Separate one‐factor RMANOVAs for each blood cannabinoid tested showed that blood plasma levels of THC and 11‐OH‐THC were significantly different across timepoints (T0, T2 and T8), and both effects were significantly quadratic (Table 3 and Figure 4). THC‐COOH did not show a significant difference across timepoints and was not found to be significantly quadratic.

**FIGURE 2:**
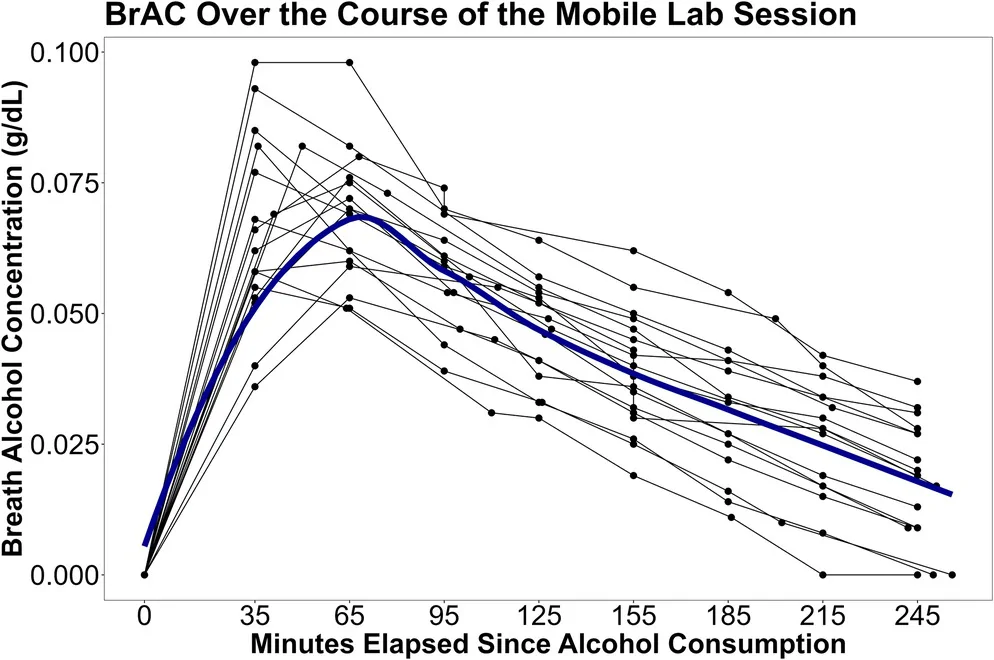
Breath alcohol concentration (BrAC) over the course of the mobile lab session. Zero minutes indicates when participants took their first sip of alcohol. BrAC measurements (black dots) are connected by black lines for each individual (*N* = 17) included in the study. A locally estimated scatterplot smoothing line for the overall sample is shown in blue.

**FIGURE 3:**
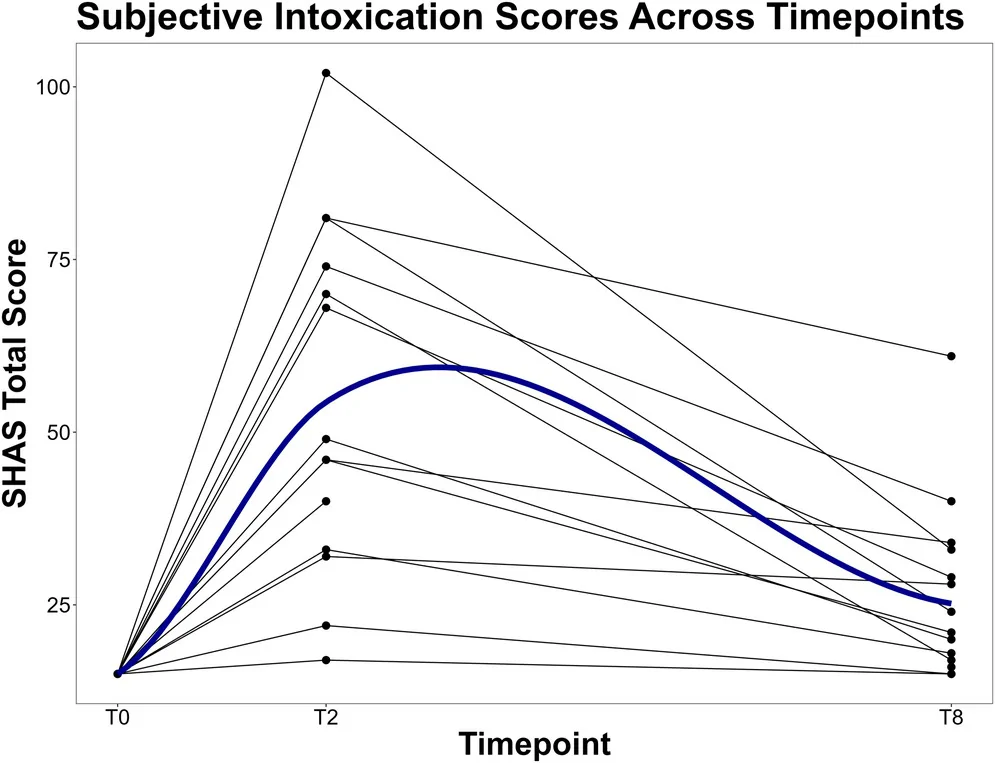
Subjective high assessment scale (SHAS) scores over the course of the mobile lab session. Individual participant (*N* = 17) scores (black dots) are connected by black lines. A locally estimated scatterplot smoothing line for the overall sample is shown in blue. *Note*. Sometimes participants did not respond to survey questions. The SHAS includes data from *n* = 14 at T2 and *n* = 16 at T8.

**FIGURE 4:**
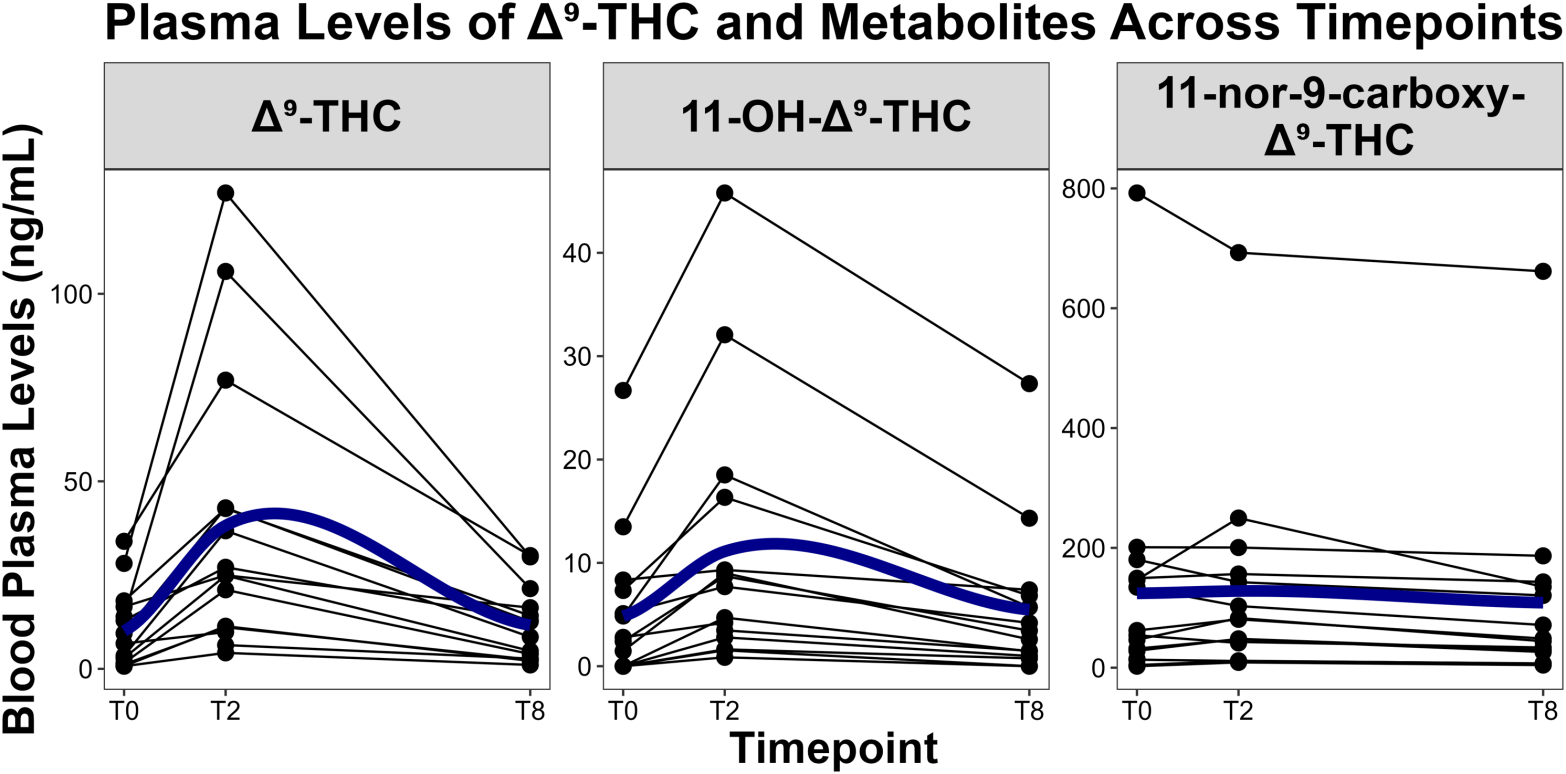
Blood plasma levels of THC and 2 THC metabolites over the course of the mobile lab session. Individual participant's Δ^9^‐THC (THC), 11‐hydroxy‐Δ^9^‐THC (11‐OH‐THC), and 11‐nor‐9‐carboxy‐Δ^9^‐THC (THC‐COOH) measurements (black dots) are connected by black lines. A locally estimated scatterplot smoothing line for the overall sample is shown in blue. *Note.* Blood plasma samples were not always successfully obtained. Across the three different cannabinoids measured, sample sizes were *n* = 15 at T0 and T2 and *n* = 14 at T8.

#### 3.1.2 Behaviours Elicited by the Flanker Task

A one‐factor RMANOVA indicated that the number of errors participants made was significantly different across timepoints, and this effect was found to be significantly quadratic (Table 3 and Figure 5A). Average post‐error slowing was also significantly different across timepoints; however, this effect was not significantly quadratic. The variability of post‐error slowing was not significantly different across timepoints. Response times (assessed using a two‐factor RMANOVA with factors timepoint: T0 vs. T2 vs. T8, and correctness of trial: correct vs. incorrect) significantly differed across timepoints, and a significant quadratic trend was observed (Table 3 and Figure 5B).

**FIGURE 5:**
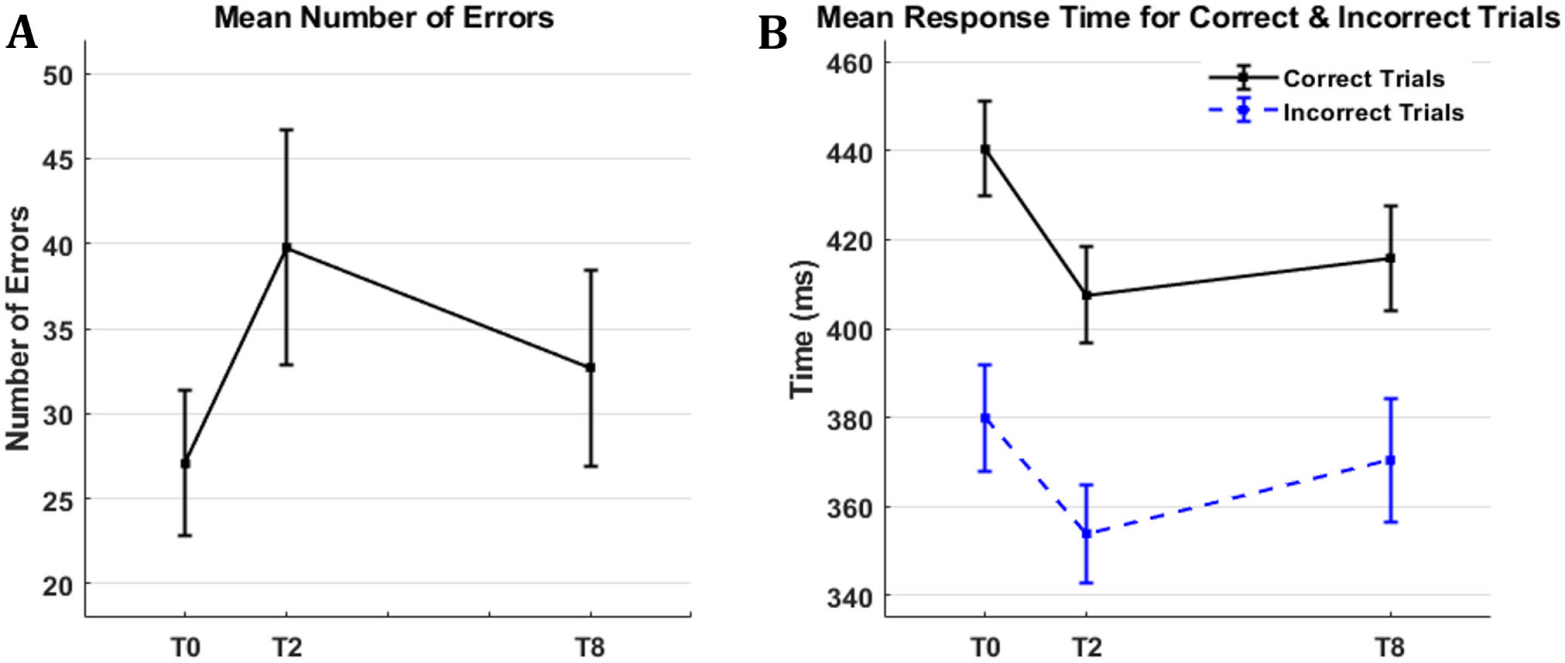
Plots of behavioural responses during the speeded flanker task conducted at the three time points; T0—sober state, T2—intoxicated state, and T8—recovery state. Subplot A shows mean number of errors with error bars representing ± standard error. Subplot B shows mean response times for correct trials (black solid line) and incorrect trials (blue dashed line) with the error bars again representing ± standard error.

### 3.2 ERP Results

Grand averaged ERPs for response‐locked, incorrect trials showing the ERN and Pe measures for the sober, intoxicated and recovery conditions are depicted in Figure 6A and associated statistics reported in Table 3 for FCz electrode. A one‐factor RMANOVA revealed that participants displayed significant differences across timepoints for both the ERN and Pe. A significant quadratic effect was observed only for the ERN (Figure 7).

**FIGURE 6:**
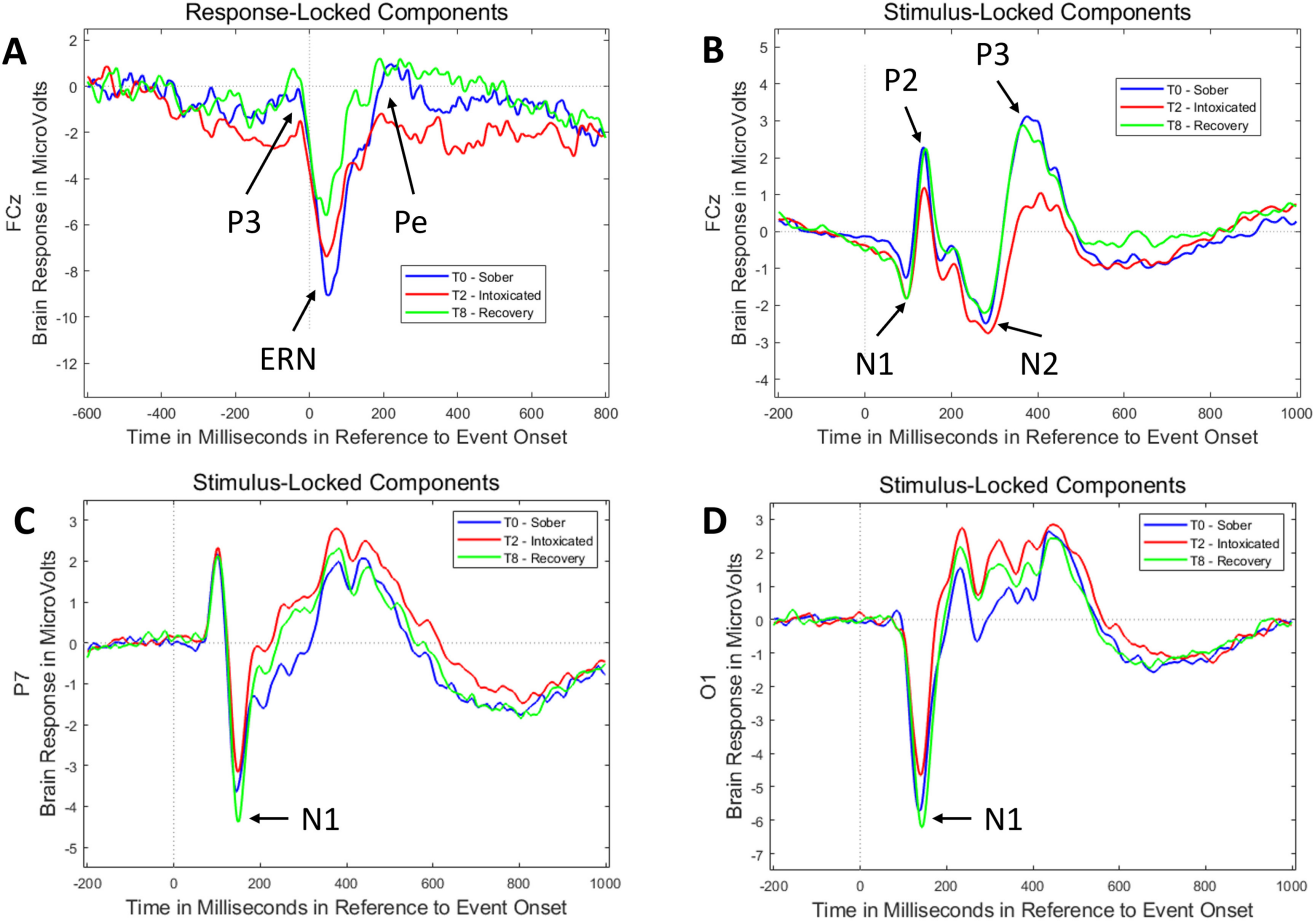
Within‐subject comparisons of ERPs from each timepoint: (A) Response‐locked grand averaged ERPs of incorrect trials at FCz, (B) stimulus‐locked grand averaged ERPs of correct trials at FCz, stimulus‐locked grand averaged ERPs of correct trials at P7 and (C) stimulus‐locked grand averaged ERPs of correct trials at O1.

**FIGURE 7:**
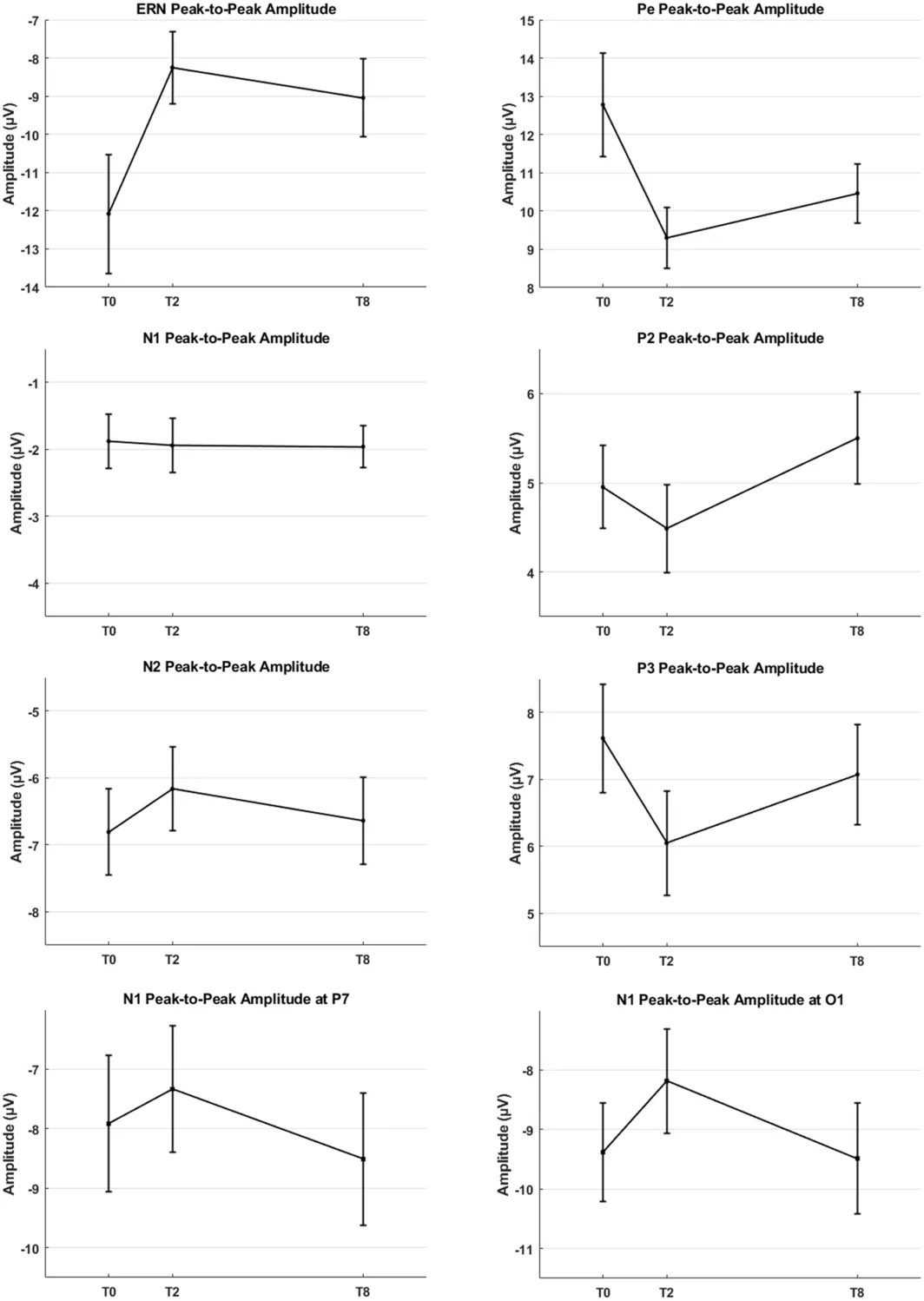
Average amplitude for response‐locked (ERN and Pe) and stimulus‐locked (N1, P2, N2 and P3) ERPs at the three time points; T0—sober state, T2—intoxicated state and T8—recovery state for FCz, except for the bottom two graphs which are for N1 at P7 and O1. The error bars represent ± standard error.

Grand averaged ERPs for stimulus‐locked, correct trials showing the N1, P2, N2 and P3 measures are depicted in Figure 6B for FCz electrode. A one‐factor RMANOVA revealed that both P2 and P3 components showed significant differences across timepoints with significant quadratic trends. The N2 component did not display a significant difference across timepoints, though it did show a significant quadratic trend. The N1 component at the frontal FCz site in the 70 to 150 ms timeframe did not display a significant main effect across timepoints nor a significant quadratic trend in the data (Figure 7). However, at the posterior electrodes, P7 and O1 in the later 130‐ to 185‐ms timeframe did display a significant difference across timepoints with significant quadratic trends.

## 4 Discussion

We investigated subjective (self‐report) and objective (behavioural, physiological, neurocognitive performance and ERPs) measures of cannabis and alcohol co‐intoxication over a 4‐h period. Subjective and objective measures of intoxication peaked 1‐h post‐substance‐consumption and showed some recovery by the end of the 4‐h period, though subjective, performance, physiological and neurophysiological measures did not return to baseline levels (i.e., full recovery did not occur). As expected, plasma THC, THC metabolites and breath alcohol levels followed a similar quadratic pattern.

Notably, self‐report ratings of intoxication differed between baseline (pre‐intoxication) and the 4‐h timepoint, indicating that some subjective intoxicating effects remained at 4‐h post‐use. However, participants displayed significantly reduced subjective intoxication from T2 to T8, and a subset of participants (*n* = 4) reported no longer feeling any substance effects by the final timepoint. Discrepancies between self‐rated intoxication and objective measures of intoxication have been previously reported in related research on driving abilities [31, 54]. These findings may reflect processes related to the ‘Mellanby effect’, in which individuals develop acute tolerance to the subjective effects of alcohol within a given drinking episode, such that subjective intoxication is greater at a given BAC when BAC is rising (i.e., during the ascending limb of the BAC curve) than at the exact same BAC when BAC is falling (i.e., during the descending limb), but objective impairment across many domains (including driving) is actually worse during the descending limb [55]. Notably, individuals are also more willing to drive during the descending limb [55], which is concerning from a public health perspective given that they still show objective impairments. Thus, the present findings have important harm‐reduction implications, as they could inform public safety messaging around the importance of avoiding driving, operating heavy machinery or engaging in otherwise risky or cognitively effortful tasks in the hours after using alcohol and cannabis, *even if the subjective intoxicating effects of these substances have decreased or worn off*.

Notably, although participants made significantly more errors on the Flanker task at T2 (and this pattern followed a quadratic trend, consistent with our second hypothesis), there was also an unexpected, significant quadratic effect on response time. The direction of this effect was opposite to our prediction, such that participants responded *faster* at T2 for correct and incorrect trials. While slower response times are typically interpreted as worse performance, this may not hold true in the context of alcohol‐related disinhibition [56, 57] where our study's results suggest more impulsive responding at T2 and T8 compared to T1. Our design involving repeated administrations of the Flanker task also introduces the potential for learning and practice effects to influence results [58]. Without a control group who remained sober throughout the 4‐h testing period, we cannot fully disentangle the effects of intoxication from the effects of fatigue and learning/practice.

Counter to hypotheses, we found that post‐error slowing decreased linearly over time (i.e., no evidence of recovery at T8), indicating a reduced ability to adjust behaviour following mistakes while intoxicated, which persists even hours later, when the subjective effects of intoxication have dissipated. Results are consistent with data suggesting that acute alcohol intoxication negatively impacts post‐error slowing [59], while cannabis administered alone has been shown to confer no effects on post‐error slowing [29, 60]. Based on these findings from previous single‐substance studies, it is possible that the observed decrements in post‐error slowing are driven by alcohol. However, the current design, which does not include alcohol‐ or cannabis‐only conditions, precludes drawing definitive conclusions about the specific contribution of each substance. Interestingly, we did not observe a significant effect of timepoint on the variability of post‐error slowing, despite a nonsignificant trend towards reduced variability over time. It is also possible that reduced post‐error slowing over time reflects learning/practice effects. Future research using larger samples could better characterize co‐intoxication effects on post‐error slowing.

Collectively, these results suggest that simultaneous alcohol and cannabis intoxication cause significant decrements in performance. It is notable that these performance decrements occurred despite the relatively low dose of alcohol given in the present study, which caused participants to reach a peak BrAC of 0.068 on average, which is below the legal limit in most states [61]. Furthermore, co‐intoxicated individuals were unable to appropriately adjust their behaviour following error commission, *and this deficit persisted even when individuals indicated feeling significantly recovered from intoxication*. This persistent inability to correct behaviour following an error could have important implications for driving, as drivers must constantly take in feedback from the environment and adjust their behaviour, particularly when an error has been made [62]. Our findings suggest that co‐intoxicated drivers—even those consuming a relatively low dose of alcohol, as in this experiment—may be unable to respond appropriately to corrective feedback while driving, and this deficit may persist even hours after substance use, when drivers no longer subjectively feel intoxicated. Further, given that participants reported feeling subjectively less intoxicated at our final assessment timepoint (~4.5 h post‐consumption) but still showed objective impairment, it will be important for future studies to examine how subjective intoxication aligns with behavioural and cognitive measures of impairment during later post‐consumption periods (e.g., 4–10 h after substance use). Including longer post‐intoxication testing timelines could help to establish how long individuals who have used alcohol and cannabis should wait before driving, given that significant impairment in cognition and behaviour can be observed even when an individual is below established BrAC thresholds and given that we lack evidence to support acceptable blood THC cutoffs for drivers [63].

Although our results broadly suggest that alcohol and cannabis co‐intoxication cause significant behavioural deficits, these substances likely confer a differential impact on each component of cognitive function, as evidenced by ERPs. We observed a significant main effect of time for P2, P3, ERN, Pe and N1 (posterior electrode sites P7 and O1 but not the frontal FCz site). The P2, N2, P3, ERN, Pe and N1 (posterior electrode sites P7 and O1 but not the frontal FCz site) also showed a significant quadratic trend. These results indicate that stimulus processing, error recognition, and post‐error adjustment‐related cognitive functions are significantly impaired by alcohol and cannabis co‐intoxication. Results are consistent with prior data suggesting that alcohol and cannabis administered separately are associated with impairments in inhibitory control and performance monitoring, including reduced ERN and Pe [28, 64] as well as P3 [17, 25], though the design of the current study precludes drawing conclusions about substance‐specific contributions to decrements in neurophysiological measures.

## 5 Limitations and Future Directions

This feasibility study should be considered as a preliminary, guiding step for future research towards understanding the neurocognitive effects of acute alcohol and cannabis co‐intoxication. Future, more hypothesis‐driven research may benefit from these initial results aimed at characterizing the neurocognitive effects of simultaneous co‐intoxication. There are several limitations inherent in our design, including the fact that due to federal laws restricting the use of legal‐market cannabis in research, we were unable to tightly control cannabis dosing. Moreover, because both substances were administered together, we cannot disentangle the independent effects of alcohol and cannabis. Future research including both single‐substance and nonintoxicated control conditions are necessary to separate the unique and potential additive/interactive contributions of each substance over time, as well as effects of fatigue and learning from the effects of intoxication over time. Also, because the sample tested included only individuals reporting heavy drinking and regular cannabis use, results may not generalize to populations who use less heavily/frequently. While the present study was conducted on a small sample size, post hoc power analyses revealed generally adequate power levels for the individual tests conducted. Additionally, while our peak‐to‐peak amplitude measurements may be more reliable than other amplitude measures (i.e., baseline‐to‐peak or average voltage), they may not be easily compared to studies which quantify ERPs using a baseline‐to‐peak or average voltage method. Also, the relatively broad age range (21–56) of participants may have contributed additional variability to the outcomes assessed. Age‐related changes in prefrontal cortical functioning, executive control and dopaminergic signalling have been associated with altered cognitive and neural activity [65, 66]. Future studies with larger samples may benefit from investigating whether acute alcohol and cannabis co‐intoxication effects vary across different ages.

Limitations associated with our ERP approach may not appropriately reflect the more dynamic cognitive processes involved in certain complex tasks (e.g., driving; [67]) and the high sensitivity of the ERP to cognitive states including fatigue, emotional state and attention [68]. However, the temporal resolution of the ERP approach allows us to distinguish nuances of cognitive function that are not identifiable with traditional behavioural/cognitive tests, making it an ideal choice for the present research question, despite methodological limitations.

## 6 Conclusions

Leveraging a unique naturalistic design, this feasibility study provides the first data on neurophysiological changes in brain activity during simultaneous alcohol and cannabis co‐intoxication. We demonstrated that EEG can be used to reliably collect lab‐grade recordings in a mobile laboratory parked at various accessible locations across the community. Furthermore, all participants wore the EEG cap and sensors for at least 4.5 consecutive hours without requesting removal, with only minimal sensor maintenance (small gel additions) required. At the intoxication timepoint, participants showed increased errors and diminished capacity to adjust behaviour following a mistake—behavioural patterns that tracked with brain activity and blood‐levels of THC, 11‐OH‐THC and alcohol. While performance and neural responses showed partial recovery by the end of the 4.5‐h study period (at which time several participants reported no longer feeling intoxicated at all), these metrics did not return to baseline levels, indicating lingering adverse behavioural and cognitive effects of intoxication despite significant subjective recovery. Lastly, the absence of significant effects on certain ERP components (e.g., N1) suggests that some cognitive processes may be less sensitive to the acute effects of simultaneous alcohol and cannabis at the intoxication levels observed in the present study.

## Author Contributions

**Eleftherios M. Hetelekides:** data curation (equal), formal analysis (equal), investigation (equal), project administration (equal), supervision (equal), validation (equal), visualization (lead), writing – original draft (lead), writing – review and editing (equal). **Patricia L. Davies:** conceptualization (equal), data curation (equal), investigation (equal), methodology (equal), project administration (equal), resources (equal), software (supporting), supervision (equal), validation (equal), writing – review and editing (equal). **Mark A. Prince:** conceptualization (equal), formal analysis (equal), funding acquisition (supporting), methodology (equal), writing – review and editing (equal). **William J. Gavin:** conceptualization (equal), data curation (equal), formal analysis (equal), investigation (lead), methodology (lead), project administration (equal), resources (equal), software (lead), supervision (equal), validation (equal), visualization (supporting), writing – review and editing (equal). **Hollis C. Karoly:** conceptualization (lead), funding acquisition (lead), investigation (equal), methodology (equal), project administration (equal), resources (equal), supervision (equal), writing – original draft (supporting), writing – review and editing (equal).

## Funding

Funding for this study was provided by the Institute of Cannabis Research Grant awarded to H.C.K. (award number ICR‐FY22‐Karoly). During the time of this study, H.C.K. was supported by the National Institute on Alcohol Abuse and Alcoholism ‐ K23AA028238.

## Ethics Statement

This research study was approved by the Institutional Review Board (IRB) at Colorado State University.

## Consent

All participants provided written, informed consent prior to participation in this research study.

## Conflicts of Interest

The authors declare no conflicts of interest.

## Permission to Reproduce Material From Other Sources

The authors have nothing to report.

## Supporting information


**Data S1:** Supporting information.

## Data Availability

The data that support the findings of this study are available from the corresponding author upon reasonable request.
