# Supplementary material for: Neurophysiological and Behavioural Effects of Combined Cannabis Concentrates and Alcohol Among People Who Regularly Use Alcohol and Cannabis
Source: Addict Biol. 2026 Oct 27;31(11):e70194. doi: 10.1111/adb.70194 (PMC13711099; doi:10.1111/adb.70194)
Supplement: Supplementary file 1 [file ADB-31-e70194-s001.docx]

**Supplemental Materials**

**Methods and Measures**

**Survey Measures**

**Measures of Past Cannabis Use.** Participants were asked to indicate the frequency of their concentrate (“On average, how often do you use cannabis concentrates (e.g. hash, oil, wax)?”) and flower (“How often do you vaporize or smoke cannabis on average?”) cannabis use and responded on a scale from “I never smoke/vaporize cannabis” to “Daily”. Participants were also asked to estimate the quantity of their flower use (“Using your best guess, what is the average amount of cannabis you use at one time?”), were presented with options from “0.1 grams” to “0.25 grams”, and were also able to write in responses.

**Beck Anxiety Inventory (BAI;** Beck et al., 1988)**.** The BAI is a self-report instrument used to assess various aspects of anxiety symptoms and includes 21-items. Participants were presented with a list of anxiety symptoms and are asked to indicate the extent to which they have been bothered by each symptom during the past week, including today. Example items include “Unable to relax” and “Nervous”. Responses are rated on a scale from 0 (“Not at all”) to 3 (“Severely - I could barely stand it.”). The range of total scores is 0 to 63 with high scores representing more anxiety. The scale showed good internal reliability, Cronbach α = 0.93.

**Beck Depression Inventory II** **(BDI-II;** Beck et al., 2011). The BDI-II (21-items) is a self-report instrument used to assess the severity of depression symptoms in a variety of populations. Participants are presented with a list of depressive symptoms and are asked to indicate the extent to which they have experienced each symptom over the past two weeks, including today. Example items include “Sadness” and “Loss of interest in activities.” Responses are rated on a scale from 0 (“I do not feel sad”) to 3 (“I am so sad or unhappy that I can't stand it”). The range of total scores is 0 to 40 with high scores representing more depression. The reliability of the scale was Cronbach α = 0.93.

**Marijuana Dependence Scale (MDS;** American Psychiatric Association, 2000)**.** The DSM-IV MDS assesses cannabis use dependence criteria. Individuals endorsing ≥3 criteria are considered to be dependent, while endorsing ≥1 is considered to be having abuse. This scale showed good internal consistency in the present study (α = 0.88).

**Alcohol Use Disorder Identification Test (AUDIT;** Babor et al., 2016)**.** The 10-item self-reported AUDIT was administered on a scale from 0-4, with summed scores above an 8 indicating potentially hazardous/harmful use. This scale showed adequate internal consistency, Cronbach α = 0.82.

**Subjective High Assessment Scale (SHAS**; Schuckit, 1985). The SHAS is a self-report scale that asks participants to examine a list of positive and negative feelings related to alcohol consumption (15-items), and indicate the extent to which each item describes their current feelings on a scale from 1 = “no alcohol effect” to 10 = “maximum alcohol effect”. Example items include “High”, “Drunk or intoxicated”, and “feelings of floating”. This measure showed strong reliability at T2 when participants were intoxicated (α = 0.92), and questionable reliability at T8 when recovering (α = 0.68).
